# Supplementary material for: A seasonal pulse of ungulate neonates influences space use by carnivores in a multi‐predator, multi‐prey system
Source: Ecol Evol. 2022 Oct 13;12(10):e9389. doi: 10.1002/ece3.9389 (PMC9558345; doi:10.1002/ece3.9389)
Supplement: Supplementary file 1 — Appendix S1 [file ECE3-12-e9389-s001.docx]

**Supporting Information**

**A seasonal pulse of ungulate neonates influences space use by carnivores in a multi-predator, multi-prey system**

Joel Ruprecht^1^, Tavis D. Forrester^2^, Nathan J. Jackson^3^, Darren A. Clark^2^, Michael J. Wisdom^4^, Mary M. Rowland^4^, Joshua B. Smith^2^, Kelley M. Stewart^3^, and Taal Levi^1^

**Table S1:** Parameter estimates for step-selection functions assessing whether coyotes, cougars, bears, and bobcats actively searched for female mule deer in the 30 days following a fawning event. The parameter “deer presence” indicates the predator’s relative probability of selection for parturient adult female mule deer by comparing whether the endpoints of real predator steps were more likely than random steps to be within a 200-meter proximity of deer. Coefficient values < 0 indicate avoidance, > 0 indicates selection, and a coefficient of 0 indicates indifference. There are no estimates for cougars and bobcats because for those species there were not enough encounters with mule deer to fit models.

|  | Coyotes | | Cougars | | Bears | | Bobcats | |
| --- | --- | --- | --- | --- | --- | --- | --- | --- |
| Parameter | Estimate | *P* | Estimate | *P* | Estimate | *P* | Estimate | *P* |
| Deer Presence | 0.28 | 0.45 | *NA* | *NA* | 0.07 | 0.84 | *NA* | *NA* |
| Canopy cover | -0.09 | <0.01 | *NA* | *NA* | 0.29 | <0.01 | *NA* | *NA* |
| Distance to road^1^ | 0.15 | <0.01 | *NA* | *NA* | 0.14 | <0.01 | *NA* | *NA* |
| Distance to water^1^ | 0.07 | 0.01 | *NA* | *NA* | -0.01 | 0.74 | *NA* | *NA* |
| Ruggedness | -0.02 | 0.53 | *NA* | *NA* | 0.04 | 0.05 | *NA* | *NA* |
| PVT, closed forest^2^ | -0.05 | 0.43 | *NA* | *NA* | 0.52 | <0.01 | *NA* | *NA* |
| PVT, open forest^2^ | -0.06 | 0.28 | *NA* | *NA* | 0.31 | <0.01 | *NA* | *NA* |
| PVT, other^2^ | -0.18 | 0.63 | *NA* | *NA* | 0.11 | 0.75 | *NA* | *NA* |
| Turn angle^3^ | -0.15 | <0.01 | *NA* | *NA* | -0.20 | <0.01 | *NA* | *NA* |
| Step length^1^ | 0.05 | <0.01 | *NA* | *NA* | 0.06 | <0.01 | *NA* | *NA* |

^1^Indicates the natural log of the variable was used. ^2^PVT = potential vegetation type, a factor variable with reference level set to “grassland.” ^3^Indicates the cosine of the variable was used.

**Table S2:** Parameter estimates for step-selection functions assessing whether male and female bears actively searched for female mule deer in the 30 days following a fawning event. The parameter “deer presence” indicates the predator’s relative probability of selection for parturient adult female deer by comparing whether the endpoints of real predator steps were more likely than random steps to be within a 200-meter proximity of deer. Coefficient values < 0 indicates avoidance, > 0 indicates selection, and a coefficient of 0 indicates indifference.

|  | Male Bears | | Female Bears | |
| --- | --- | --- | --- | --- |
| Parameter | Estimate | *P* | Estimate | *P* |
| Deer Presence | 0.06 | 0.88 | 0.16 | 0.88 |
| Canopy cover | 0.27 | <0.01 | 0.33 | <0.01 |
| Distance to road^1^ | 0.12 | <0.01 | 0.23 | <0.01 |
| Distance to water^1^ | -0.01 | 0.48 | 0.01 | 0.76 |
| Ruggedness | -0.02 | 0.50 | 0.12 | <0.01 |
| PVT, closed forest^2^ | 0.57 | <0.01 | 0.48 | <0.01 |
| PVT, open forest^2^ | 0.37 | <0.01 | 0.23 | 0.02 |
| PVT, other^2^ | 0.17 | 0.68 | -0.04 | 0.96 |
| Turn angle^3^ | -0.26 | <0.01 | -0.08 | 0.02 |
| Step length^1^ | 0.05 | <0.01 | 0.08 | <0.01 |

^1^Indicates the natural log of the variable was used. ^2^PVT = potential vegetation type, a factor variable with reference level set to “grassland.” ^3^Indicates the cosine of the variable was used.

**Table S3:** Parameter estimates for step-selection functions assessing whether coyotes, cougars, bears, and bobcats actively searched for female elk in the 30 days following a calving event. The parameter “elk presence” indicates the predator’s relative probability of selection for parturient adult female elk by comparing whether the endpoints of real predator steps were more likely than random steps to be within a 200-meter proximity of elk. Coefficient values < 0 indicates avoidance, > 0 indicates selection, and a coefficient of 0 indicates indifference.

|  | Coyotes | | Cougars | | Bears | | Bobcats | |
| --- | --- | --- | --- | --- | --- | --- | --- | --- |
| Parameter | Estimate | *P* | Estimate | *P* | Estimate | *P* | Estimate | *P* |
| Elk Presence | -0.15 | 0.45 | 1.11 | <0.01 | 0.31 | 0.06 | -0.59 | 0.20 |
| Canopy cover | -0.09 | <0.01 | 0.23 | <0.01 | 0.30 | <0.01 | 0.31 | <0.01 |
| Distance to road^1^ | 0.10 | <0.01 | 0.03 | 0.08 | 0.11 | <0.01 | 0.07 | 0.04 |
| Distance to water^1^ | 0.05 | 0.01 | -0.10 | <0.01 | 0.01 | 0.21 | 0.07 | 0.04 |
| Ruggedness | -0.04 | 0.02 | 0.19 | <0.01 | 0.06 | <0.01 | 0.04 | 0.14 |
| PVT, closed forest^2^ | -0.07 | 0.17 | 0.51 | <0.01 | 0.38 | <0.01 | 1.27 | <0.01 |
| PVT, open forest^2^ | -0.04 | 0.31 | 0.29 | <0.01 | 0.22 | <0.01 | 0.97 | <0.01 |
| PVT, other^2^ | -0.27 | 0.33 | 0.75 | <0.01 | 0.17 | 0.53 | -11.72 | 0.99 |
| Turn angle^3^ | -0.13 | <0.01 | -0.08 | <0.01 | -0.26 | <0.01 | 0.01 | 0.78 |
| Step length^1^ | 0.05 | <0.01 | 0.05 | <0.01 | 0.04 | <0.01 | 0.04 | <0.01 |

^1^Indicates the natural log of the variable was used. ^2^PVT = potential vegetation type, a factor variable with reference level set to “grassland.” ^3^Indicates the cosine of the variable was used.

**Table S4:** Parameter estimates for step-selection functions assessing whether male and female bears actively searched for female elk in the 30 days following a calving event. The parameter “elk presence” indicates the predator’s relative probability of selection for parturient adult female elk by comparing whether the endpoints of real predator steps were more likely than random steps to be within a 200-meter proximity of elk. Coefficient values < 0 indicates avoidance, > 0 indicates selection, and a coefficient of 0 indicates indifference.

|  | Male Bears | | Female Bears | |
| --- | --- | --- | --- | --- |
| Parameter | Estimate | *P* | Estimate | *P* |
| Elk Presence | 0.56 | <0.01 | -0.45 | 0.24 |
| Canopy cover | 0.27 | <0.01 | 0.34 | <0.01 |
| Distance to road^1^ | 0.09 | <0.01 | 0.19 | <0.01 |
| Distance to water^1^ | <0.01 | 0.99 | 0.04 | 0.04 |
| Ruggedness | 0.01 | 0.74 | 0.13 | <0.01 |
| PVT, closed forest^2^ | 0.37 | <0.01 | 0.41 | <0.01 |
| PVT, open forest^2^ | 0.18 | <0.01 | 0.30 | <0.01 |
| PVT, other^2^ | 0.01 | 0.97 | 0.52 | 0.28 |
| Turn angle^3^ | -0.33 | <0.01 | -0.09 | <0.01 |
| Step length^1^ | 0.03 | <0.01 | 0.07 | <0.01 |

^1^Indicates the natural log of the variable was used. ^2^PVT = potential vegetation type, a factor variable with reference level set to “grassland.” ^3^Indicates the cosine of the variable was used.

**Text S1: Derivation of the profitability of two prey resources for a rate-maximizing predator.**

The energetic profitability (measured in energy per unit time) of a species upon encounter is given by *Ei* /*hi* , where *Ei*  and *hi* are the energy content and handling time of species *i* respectively. If we consider species 1 to be herbivore neonates and species 2 to be other prey resources, then assuming that herbivore neonates are more energetically profitable upon encounter leads to *E*1 /*h*1 > *E*2 /*h*2. This suggests that a rate maximizing predator should specialize on neonates, but of course ignoring species 2 introduces the time cost of searching for species 1, *S*1. The inequality for specializing on species 1 then becomes,

$$\frac{E_{1}}{h_{1}+S_{1}}>\frac{E_{2}}{h_{2}}$$

such that a sufficiently low search time for species 1, such as during a pulse of neonates, could leads to specialization which would manifest in searching behavior rather than incidental encounter. This can be seen explicitly by solving for *S*1, which yields the inequality that specialization should occur as long as the search time for species 1 follows

$$S_{1}<\frac{{h_{2}E}_{1}}{E_{2}}-h_{1}$$

As species 2 becomes more profitable ($E_{2}$ increases or $h_{2}$ declines) or the handling time of species 1 increases, the search time must be smaller (or encounter rate larger) to make specialization an optimal foraging strategy.

**
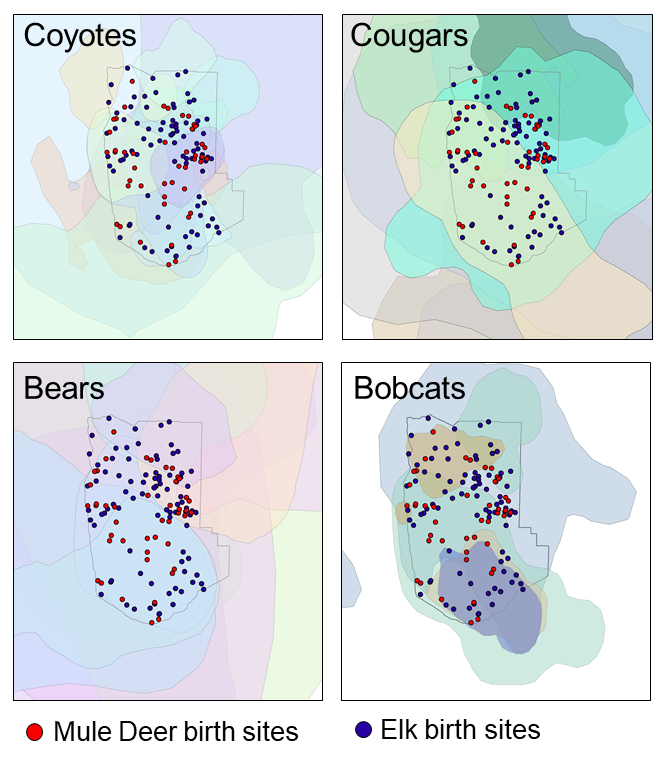
**

**Figure S1:** Home range estimates (based on 95% kernel density isopleths) for GPS-collared carnivores. Each polygon represents a different individual, and each home range is presented at 50% transparency to display multiple overlapping individuals. Mule deer birth sites are shown by red points and elk birth sites are shown by blue points.

**
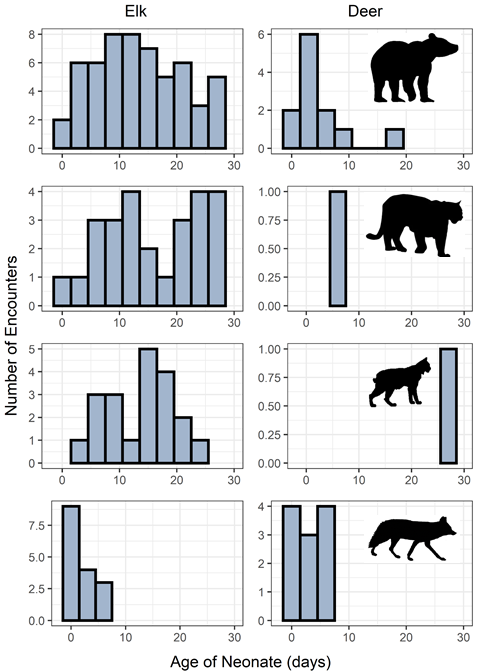
**

**Figure S2:** The number of encounters of GPS-collared carnivores within a radius of 200m of GPS-collared parturient prey as a function of the time since the birth of the encountered neonate. Rows from top to bottom display encounters by black bears, cougars, bobcats, and coyotes, respectively.


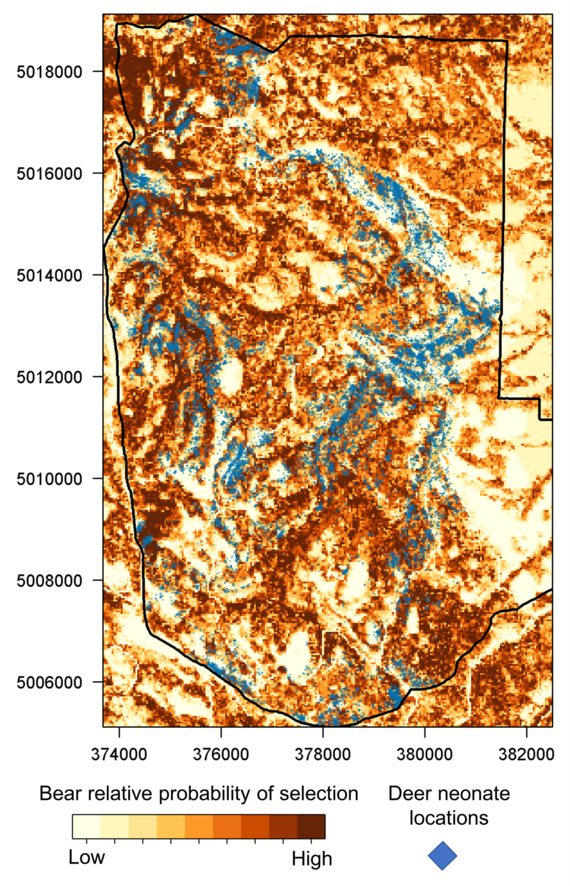


**Figure S3**: Relative probability of selection for black bears (male and female) predicted from step selection functions where darker shades of brown indicate higher relative probability of selection. GPS locations of telemetered adult female mule deer in the 30 days post-parturition (blue points) are overlaid on the relative probability of selection map. Deer locations appear darker when they overlap pixels with higher (i.e. darker shades of) relative probabilities of selection for bears.


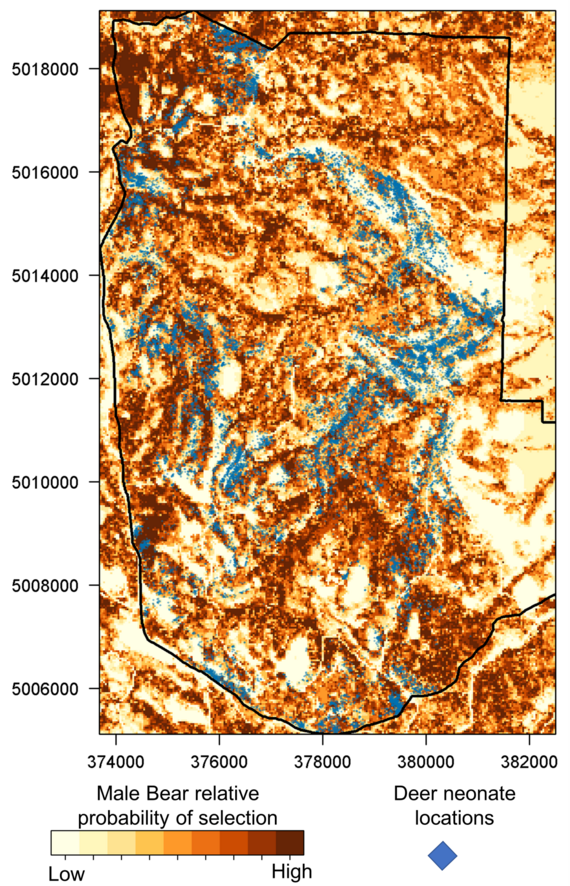


**Figure S4**: Relative probability of selection for male black bears predicted from step selection functions where darker shades of brown indicate higher relative probability of selection. GPS locations of telemetered adult female mule deer in the 30 days post-parturition (blue points) are overlaid on the relative probability of selection map. Deer locations appear darker when they overlap pixels with higher (i.e. darker shades of) relative probabilities of selection for male bears.


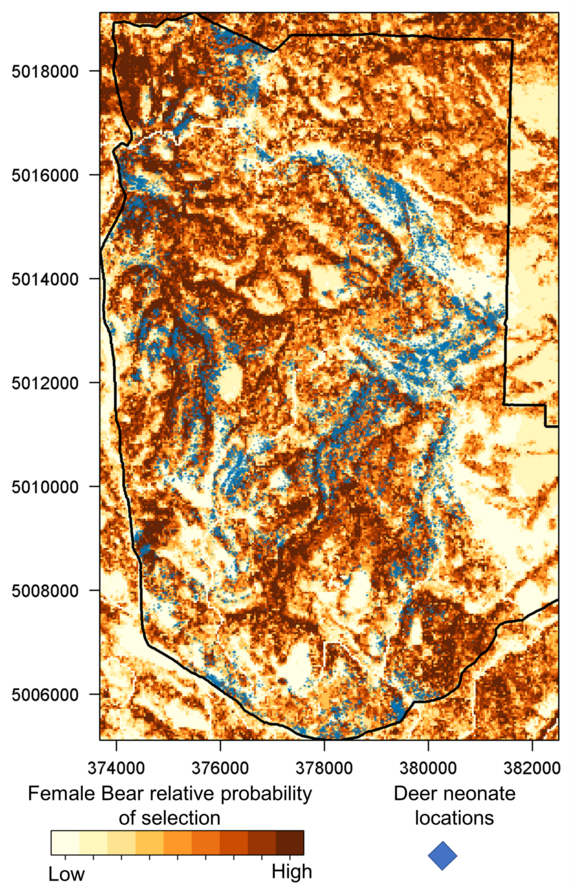


**Figure S5**: Relative probability of selection for female black bears predicted from step selection functions where darker shades of brown indicate higher relative probability of selection. GPS locations of telemetered adult female mule deer in the 30 days post-parturition (blue points) are overlaid on the relative probability of selection map. Deer locations appear darker when they overlap pixels with higher (i.e. darker shades of) relative probabilities of selection for female bears.


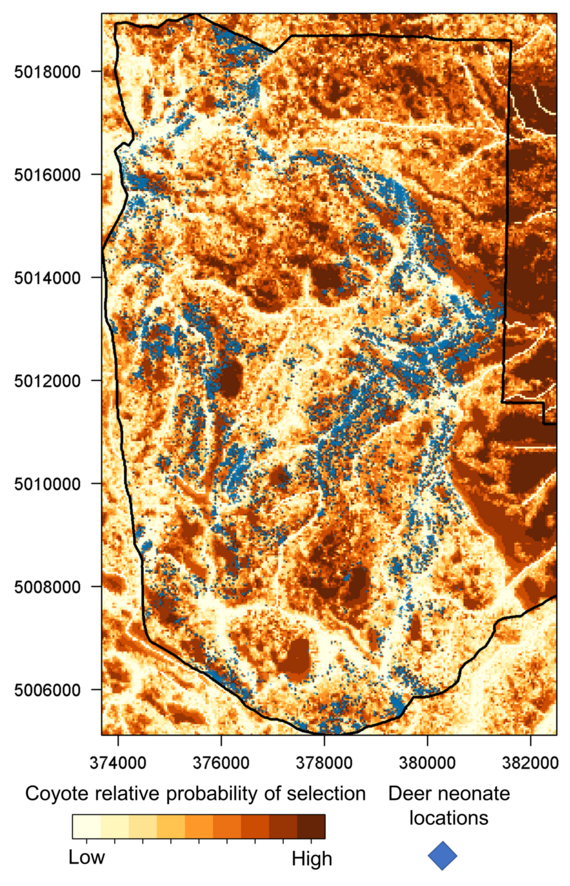


**Figure S6**: Relative probability of selection for coyotes predicted from step selection functions where darker shades of brown indicate higher relative probability of selection. GPS locations of telemetered adult female mule deer in the 30 days post-parturition (blue points) are overlaid on the relative probability of selection map. Deer locations appear darker when they overlap pixels with higher (i.e. darker shades of) relative probabilities of selection for coyotes.


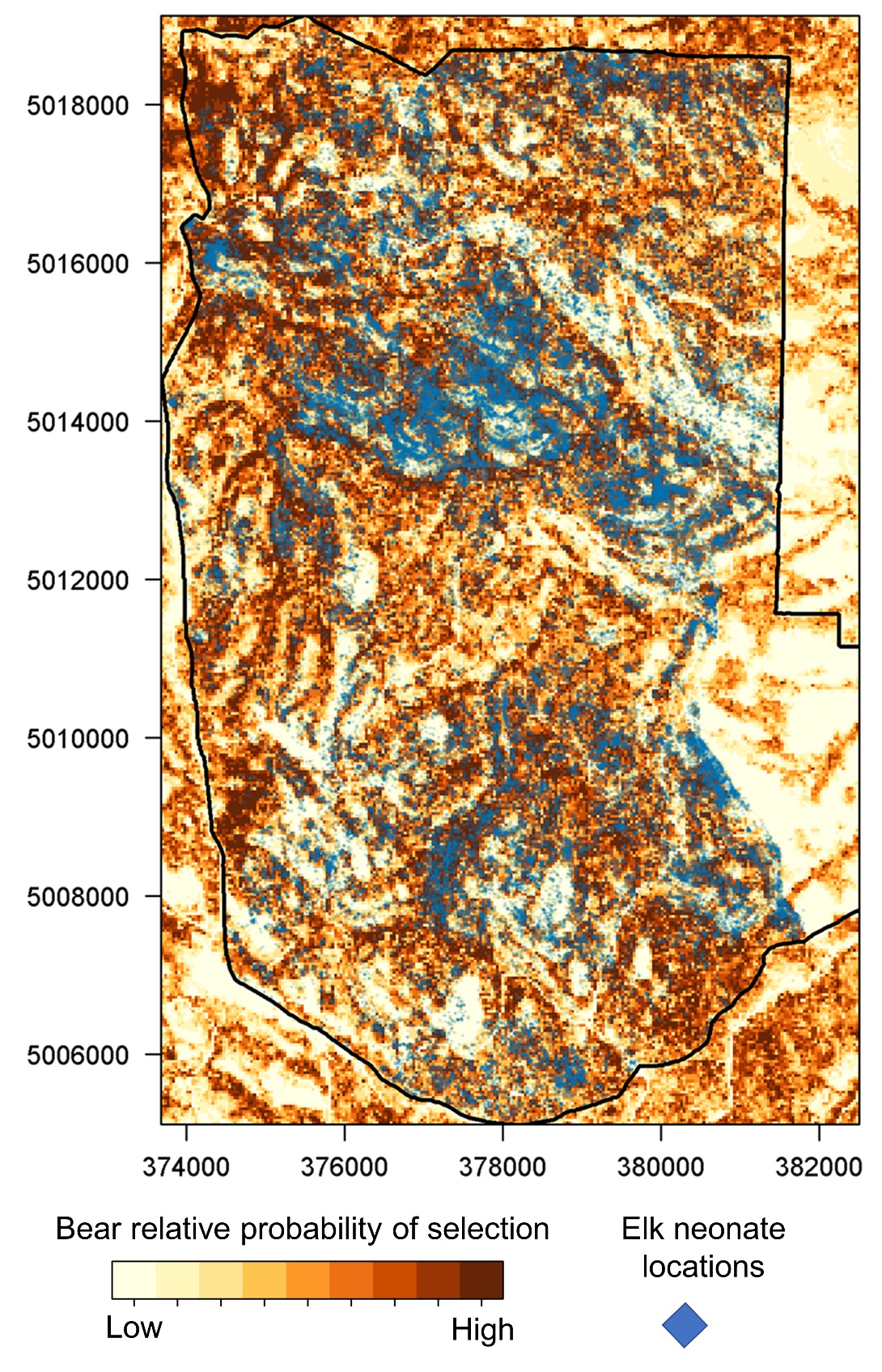


**Figure S7**: Relative probability of selection for bears (male and female) predicted from step selection functions where darker shades of brown indicate higher relative probability of selection. GPS locations of telemetered adult female elk in the 30 days post-parturition (blue points) are overlaid on the relative probability of selection map. Elk locations appear darker when they overlap pixels with higher (i.e. darker shades of) relative probabilities of selection for bears.


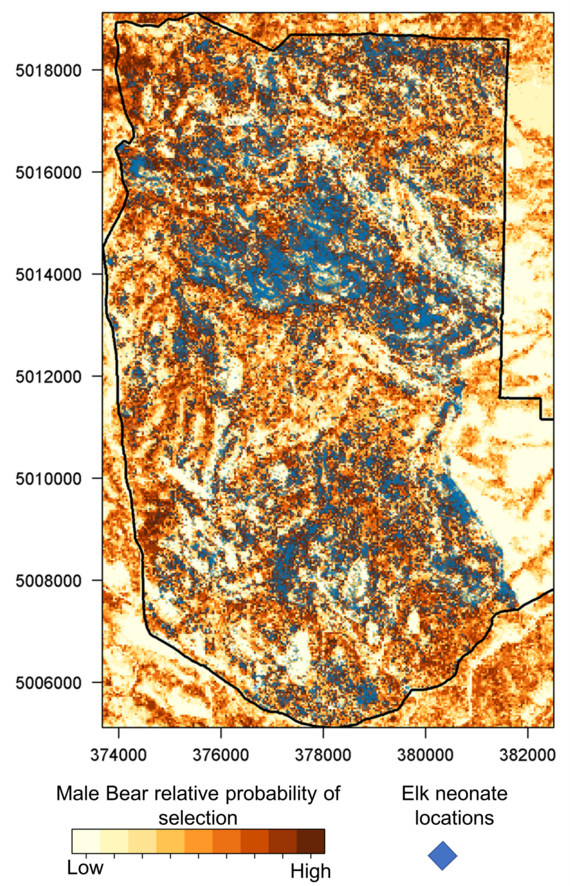


**Figure S8**: Relative probability of selection for male bears predicted from step selection functions where darker shades of brown indicate higher relative probability of selection. GPS locations of telemetered adult female elk in the 30 days post-parturition (blue points) are overlaid on the relative probability of selection map. Elk locations appear darker when they overlap pixels with higher (i.e. darker shades of) relative probabilities of selection for male bears.


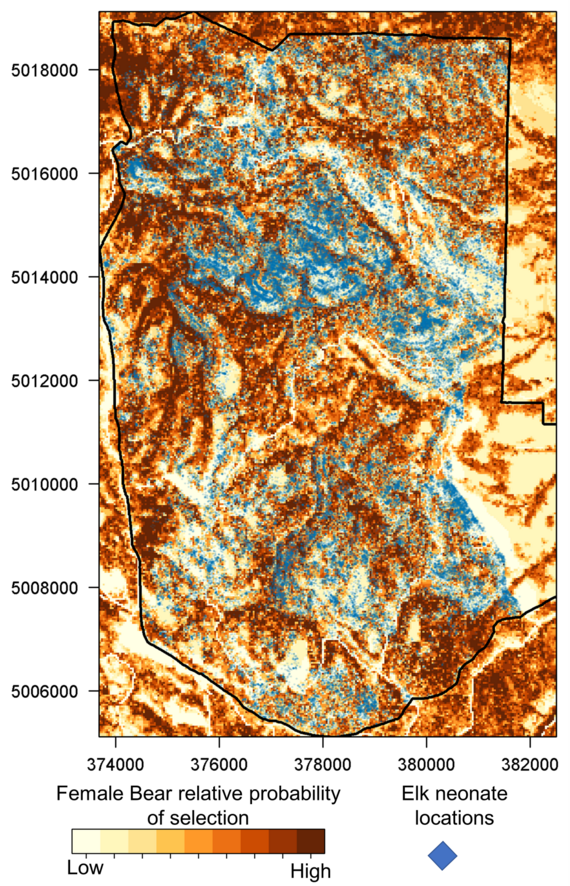


**Figure S9**: Relative probability of selection for female bears predicted from step selection functions where darker shades of brown indicate higher relative probability of selection. GPS locations of telemetered adult female elk in the 30 days post-parturition (blue points) are overlaid on the relative probability of selection map. Elk locations appear darker when they overlap pixels with higher (i.e. darker shades of) relative probabilities of selection for female bears.


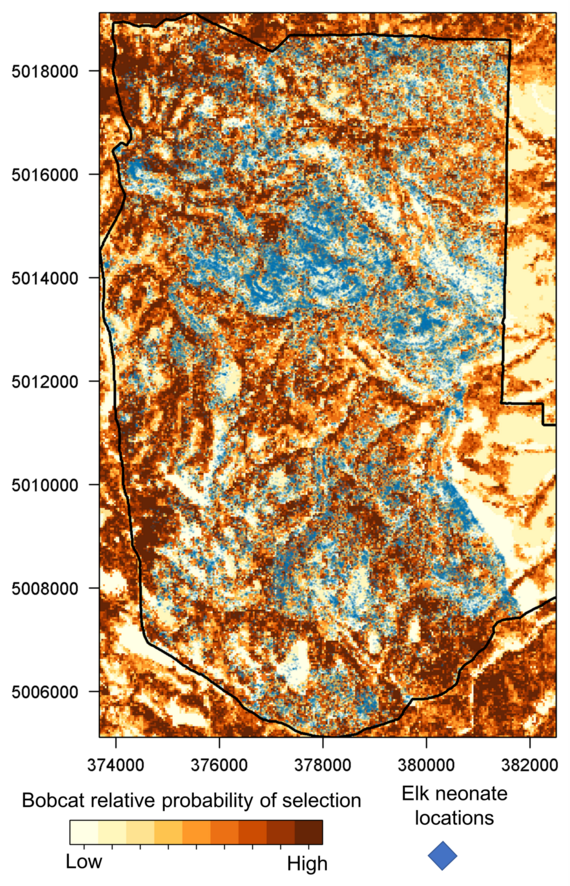


**Figure S10**: Relative probability of selection for bobcats predicted from step selection functions where darker shades of brown indicate higher relative probability of selection. GPS locations of telemetered adult female elk in the 30 days post-parturition (blue points) are overlaid on the relative probability of selection map. Elk locations appear darker when they overlap pixels with higher (i.e. darker shades of) relative probabilities of selection for bobcats.


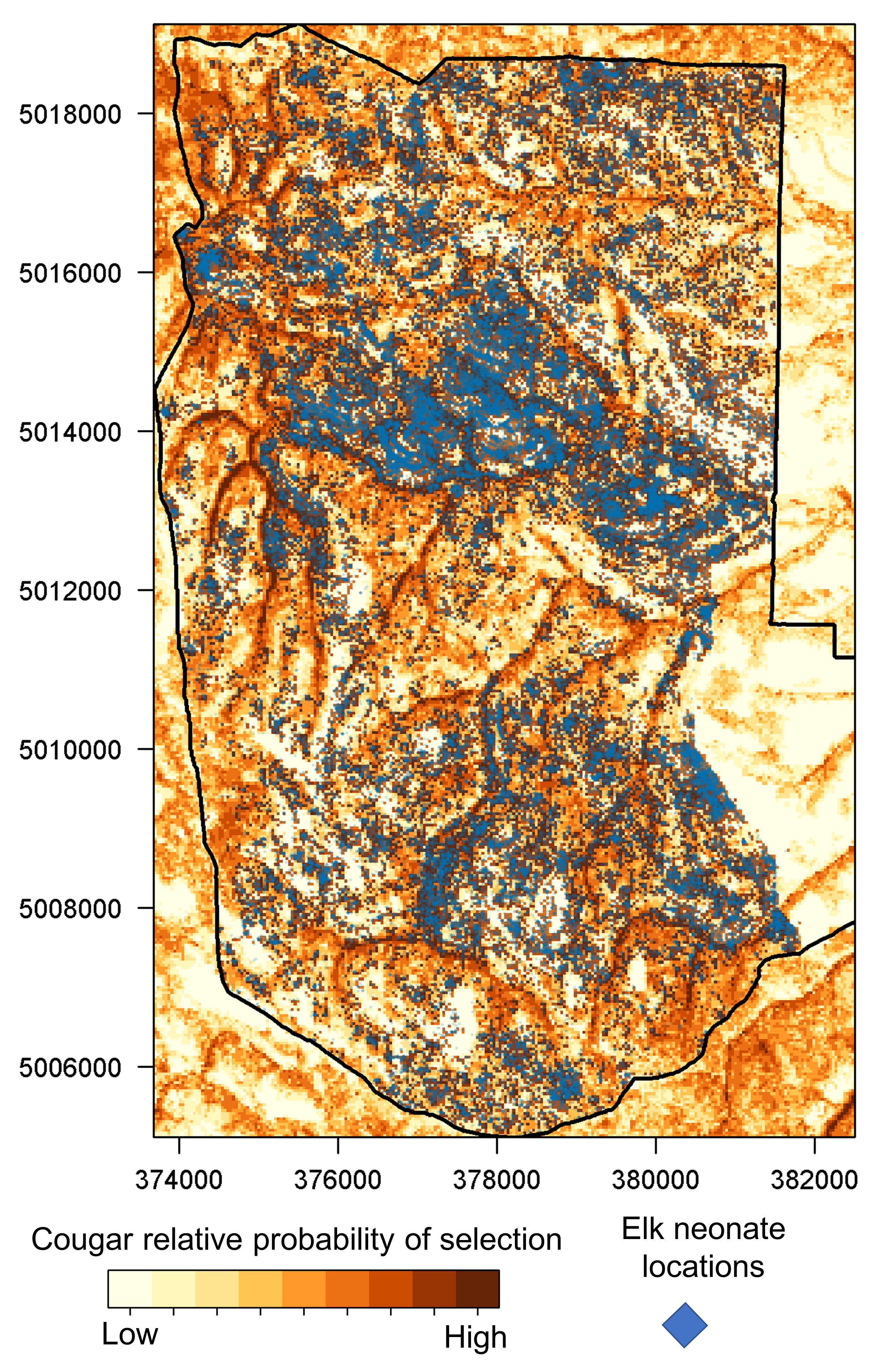


**Figure S11**: Relative probability of selection for cougars predicted from step selection functions where darker shades of brown indicate higher relative probability of selection. GPS locations of telemetered adult female elk in the 30 days post-parturition (blue points) are overlaid on the relative probability of selection map. Elk locations appear darker when they overlap pixels with higher (i.e. darker shades of) relative probabilities of selection for cougars.


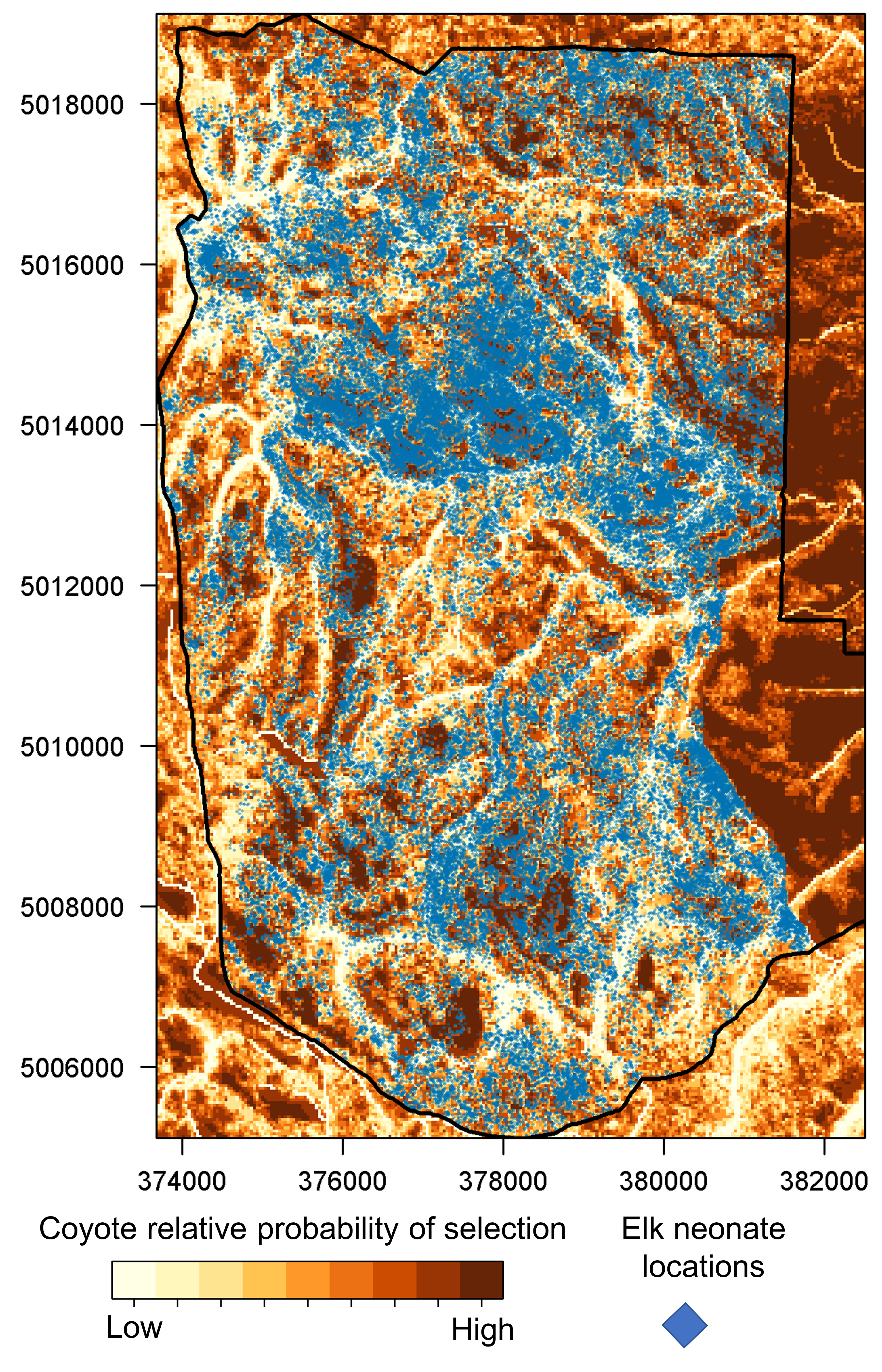


**Figure S12**: Relative probability of selection for coyotes predicted from step selection functions where darker shades of brown indicate higher relative probability of selection. GPS locations of telemetered adult female elk in the 30 days post-parturition (blue points) are overlaid on the relative probability of selection map. Elk locations appear darker when they overlap pixels with higher (i.e. darker shades of) relative probabilities of selection for coyotes.
